# The Impact of Injector-Based Contrast Agent Administration on Bolus Shape and Magnetic Resonance Angiography Image Quality

**DOI:** 10.1177/1178623x17705894

**Published:** 2017-04-20

**Authors:** Gregor Jost, Jan Endrikat, Hubertus Pietsch

**Affiliations:** 1 MR and CT Contrast Media Research, Bayer AG, Berlin, Germany; 2 Global Medical & Clinical Affairs Radiology, Bayer AG, Berlin, Germany; 3 Department of Gynecology, Obstetrics and Reproductive Medicine, University Medical School of Saarland, Homburg, Germany

**Keywords:** Magnetic resonance imaging, magnetic resonance angiography, contrast agent, power injector, gadobutrol

## Abstract

**Objective:**

To compare injector-based contrast agent (CA) administration with hand injection in magnetic resonance angiography (MRA).

**Methods:**

Gadobutrol was administered in 6 minipigs with 3 protocols: (a) hand injection (one senior technician), (b) hand injection (6 less-experienced technicians), and (c) power injector administration. The arterial bolus shape was quantified by test bolus measurements. A head and neck MRA was performed for quantitative and qualitative comparison of signal enhancement.

**Results:**

A significantly shorter time to peak was observed for protocol C, whereas no significant differences between protocols were found for peak height and bolus width. However, for protocol C, these parameters showed a much lower variation. The MRA revealed a significantly higher signal-to-noise ratio for injector-based administration. A superimposed strong contrast of the jugular vein was found in 50% of the hand injections.

**Conclusions:**

Injector-based CA administration results in a more standardized bolus shape, a higher vascular contrast, and a more robust visualization of target vessels.

## Introduction

In contrast-enhanced magnetic resonance angiography (CE-MRA), luminograms are obtained during the first arterial passage of gadolinium-based contrast agents (GBCAs).^1^ Therefore, the vascular bolus geometry (ie, the bolus width and peak height) and timing between the arrival of the contrast bolus and the magnetic resonance angiography (MRA) image acquisition have an important impact on vascular signal enhancement and hence the image contrast. Over the past 2 decades, magnetic resonance imaging (MRI) hardware improvements, parallel imaging, and the use of advanced *k*-space sampling methods, such as elliptic centric and spiral acquisitions, greatly enhanced the MRA image quality by increasing the spatial resolution and reducing the acquisition time.^2–4^ However, accurate timing between GBCA injection and image acquisition is required for all 3-dimensional (3D)-MRA techniques. If the *k*-space center is not sampled during the arterial bolus peak, the vascular contrast is insufficient. In addition, if sampling starts are delayed, then the arterial phase is overlaid with venous signal enhancements. Moreover, artifacts such as vessel broadening and edge ringing are reported for suboptimal timing.^5^ Synchronization for bolus arrival and image acquisition can be ensured using bolus tracking techniques or prior test bolus measurements.^6–8^

In addition to the correct timing, the vascular bolus geometry has to be adapted to the diagnostic objective (ie, the vascular structure to be enhanced) and MRA sequence settings, particularly the scan duration.^5,9^ The bolus geometry depends on the injection protocol, patient-specific parameters (eg, cardiac output),^10^ and the GBCA concentration.^11^ Considering the approved GBCA dose of 0.1 mmol/kg body weight (for most of the GBCA), the injection rate and volume of the saline flush are parameters that should be optimized.^12–14^ A higher injection rate results in a higher vascular bolus peak and thus an increased signal-to-noise ratio (SNR).^15^ In contrast, the resulting shorter injection time requires more precise timing and is more prone to edge-ringing artifacts if the bolus concentration changes too rapidly during the acquisition of central *k*-space lines.^5^ A well-controlled GBCA injection procedure with respect to bolus timing and the injection rate is critical for all CE-MRA techniques.

In Germany, 62.1% of all MRA procedures involve the use of a power injector for CA administration, and in the Unites States, in the first half of 2016, 72.7% of MRA procedures are run with a power injector.^16^ Thus, hand injections are frequently used, although they are operator dependent and have no control over the precise injection rate. In addition, a temporal gap between the administration of GBCA and saline exists depending on the tubing set used. The use of a 3-way stopcock in combination with a contrast and saline syringe enables nearly successive injections, whereas a larger time delay may exist when changing syringes directly on the tubing. In contrast, automated GBCA administration using power injectors enables the successive administration of GBCA and saline under highly controlled conditions. Although the benefits of injector-based administration seem to be apparent, the experimental evidence is very limited. Only a few studies exist about the technical aspects of CA administration.^15,17,18^ In fact, to our knowledge, no systematic comparison between hand injection and injector-based CA injection in MRA has been published so far.

The aim of this study was to evaluate the impact of automated, injector-based GBCA administration on the vascular bolus shape and MRA image quality compared with hand injection. Therefore, test bolus profiles and quantitative and qualitative MRA signal evaluations were compared in an animal model under standardized conditions.

## Materials and Methods

### Animals

Six Göttingen minipigs (2 females, 4 males, 27.8 ± 3.6 kg body weight) were examined in a 1.5-T clinical whole-body MRI. The animals were handled in compliance with the German animal welfare legislation and approval of the state animal welfare committee. All studies were performed under general anesthetic induced with ketamine, 30 mg/kg body weight intramuscular (Pharmacia, Karlsruhe, Germany); azaperone, 2 mg/kg body weight intramuscular (Stresnil; Janssen-Cilag, Neuss, Germany); and atropine, 0.025 mg/kg body weight (Eifelfango Chem.-Pharm. Werke, Bad Neuenahr-Ahrweiler, Germany). After intravenous application of 1.4 mg/kg propofol (Propofol-ratiopharm; Ratiopharm, Ulm, Germany), the animals were orally intubated (Roesch tube 6.0; Teleflex Medical, Kernen, Germany) and mechanically ventilated with an oxygen-air-mixture using an anesthesia workstation (Servo Ventilator 900C; Siemens, Erlangen, Germany). Anesthesia was maintained by intravenous injection with propofol (0.8 mg/kg/h). Animals were placed in a prone position, and the heart rate was monitored before each contrast administration. Test bolus and MRA imaging was performed during end-expiratory breath hold.

### Contrast protocols

Gadobutrol (Gadovist; Bayer Vital GmbH, Leverkusen, Germany) was administered intravenously into an ear vein using a 20-gauge access. To obtain an intra-individual comparison, each animal received 3 contrast injections (protocols A, B, and C) in a randomized order with a resting time of at least 60 minutes between consecutive injections. For each protocol, a standard dose of 0.1 mmol/kg body weight was used including a 0.5 mL test bolus. All injections were followed by a 20-mL saline chaser. In protocols A and B, GBCA administration was performed manually by hand injection. Therefore, 5 and 20 mL syringes (Omnifix; Braun Melsungen, Melsungen, Germany) were used together with saline-prefilled patient tubing. In combination with a 3-way stopcock (Discofix; Braun Melsungen), this setup was used to inject GBCA and saline in a successive manner. In protocol C, GBCA was administered with a dual-head power injector (Medrad Spectris Solaris; Bayer US, Indianola, PA, USA) using a flow rate of 2 mL/s for GBCA and saline. In protocol A, contrast injection was performed by an experienced senior technician who has performed more than 1000 hand injections. In protocol B, injections were performed by 6 technicians with less experience. All technicians were asked to inject at a flow rate of 2 mL/s after a receiving a start command from the MRI scanner operator.

### MRI protocol

MRI imaging was performed in a clinical 1.5-T MRI (Avanto; Siemens Healthcare, Erlangen, Germany) using a spine coil in combination with a head and neck coil. For the test bolus, a single slice FLASH sequence (repetition time [TR] = 35 ms, echo time [TE] = 1.43 ms, α = 30°) with a slice thickness of 18 mm with parallel flow saturation pulses was orthogonally adjusted to the carotid arteries (A. carotis communis). The scan was simultaneously started with the contrast injection, and 50 measurements were performed with a temporal resolution of 1 s. The MRA scan delay time (*t_delay_*) was calculated based on the time-to-peak (TTP) analysis performed with Mean Curve software (Siemens Healthcare):

$$t_{delay}=TTP-t_{k-space\;center}+0.5t_{injection}$$

In this equation, the injection time (*t_injection_*) corresponds to the individual duration of the GBCA administration with 2 mL/s. The time to the *k*-space center (*t_k-space center_*) was 7.1 s with the used linear ordering of the MRA sequence (VIBE, TR = 3.05 ms, TE = 1.06 ms, α = 25°, GRAPPA = 2). An isotropic spatial resolution of 1.2 mm was used with an imaging matrix of 168 × 384 × 112 pixels. The resulting scan time was 19 seconds. Magnetic resonance angiography was performed in the coronal orientation to cover the head, neck, and thorax until the aortic arch. A baseline scan was acquired twice with the first as a subtraction mask and the second for noise calculations followed by the CE-MRA.

### Image evaluation

The test bolus profile was evaluated for each individual test bolus curve by quantification of the bolus height and full width at half maximum (FWHM). For the FWHM analysis, a gamma variate fit was applied to the curves using a MATLAB script (Matlab 7.1; MathWorks, Natick, MA, USA).^19^ The bolus timing was quantified by TTP analysis and calculation of a second time period; the rise time (RT) was defined as the time from contrast arrival to the peak:

RT=TTP−TTA

where *TTA* was the time from injection until an increase in signal intensity ⩾10 (arbitrary units) in the fitted bolus curves.

Quantitative analysis of the MRA signal enhancement was performed on the subtraction images in regions of interest located in 7 representative arteries: ascending aorta, descending aorta, truncus brachiocephalicus, A. carotis communis, A. carotis externa, A. maxillaris, and A. lingualis. The last 4 arteries were evaluated dexter and sinister. Signal-to-noise ratio (SNR) calculations were performed by the method suggested by Reeder et al.^20^ Therefore, the MRA signal intensity *S_MRA_* was divided by the standard deviation (*std*) of the signal intensity difference from 2 baseline scans (*S_bl_*_1_, *S_bl_*_2_):

$$SNR=\frac{S_{MRA}}{\sqrt{2}\cdot std(S_{bl1}-S_{bl2})}$$

Qualitative evaluation of the vascular contrast was performed using maximum intensity projections (MIP). The criteria for image evaluation included visualization of the target vessels in the head and neck region. High contrast was defined as high vascular signal intensity with clear vessel delineation, and low contrast was defined as nonuniform visualization of vessel segments with signal intensities near the background level.

### Statistical evaluation

All values are presented as mean ± standard deviation; *p* values are reported for all statistically significant differences. For each parameter analyzed, the coefficient of variation (mean value divided by standard deviation) was calculated to compare variations between the animals in protocols A, B, and C. Statistical comparisons between the 3 protocols were performed using 1-way repeated-measures analysis of variance (heart rate, peak enhancement, FWHM, TTP, and RT). For the SNR analysis, 2-way repeated-measures analyses of variance with vessel and contrast protocol as factors were used. Both procedures were followed by the post-hoc Tukey test for multiple group comparison. The calculations were performed with GraphPad Prism (GraphPad Software, La Jolla, CA, USA) using a significance level of 5%.

## Results

All 3 MRA examinations were successfully performed with all 6 animals. The heart rate showed no significant differences between protocols A, B, and C, which had rates of 104.4 ± 19.2, 106 ± 22.8, and 107 ± 15.5 bpm, respectively.

### Test bolus curves

**Figure 1.**
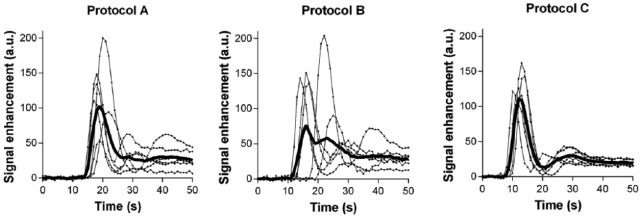
Test bolus curves for injection protocols A, B, and C. The averaged bolus curves are visualized by the thick lines; individual curves for each animal are shown as thin lines.

An averaged test bolus curve for hand injection protocols A and B showed a broader bolus width with lower peak signal intensity and a longer TTP than that of automatic injection (protocol C, Figure 1). In contrast to protocol A (injection by 1 experienced technician) and protocol C (injector), the bolus curve for protocol B showed an inconsistent average bolus shape due to the individual variation of the 6 different technicians.

### Quantitative analysis of bolus shape

**Figure 2.**
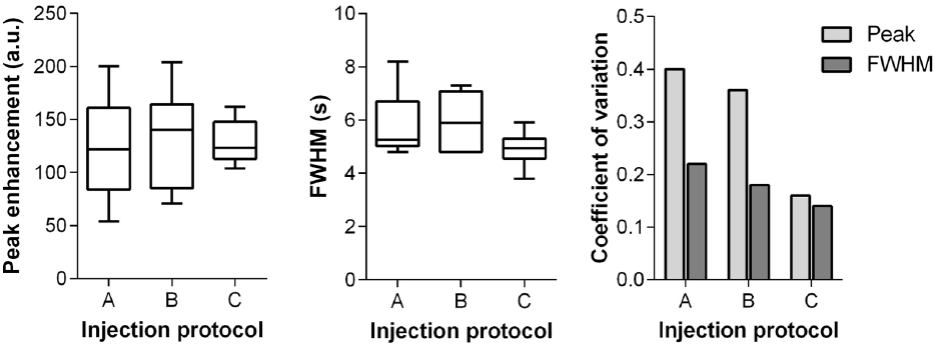
Quantitative evaluation of test bolus shape and parameter variations in injection protocols A, B, and C: peak enhancement (left), full width at half maximum (FWHM, middle) and coefficient of variation (right).

The quantitative parameters based on the individual test bolus curves for protocols A, B, and C are summarized in Table 1. The peak signal intensities reveal comparable mean values with no significant differences between the 3 protocols. However, the variability of the peak signals quantified by the coefficient of variation is lower for protocol C than that for protocols A and B (Figure 2). The lowest bolus FWHM was observed for protocol C followed by protocol A and then B. Again, the lowest variation was observed for protocol C (Figure 2).

**Table 1.** Quantitative test bolus–derived parameters: peak signal intensity (peak), full width at half maximum (FWHM), time to peak (TTP), and rise time (RT) for injection protocols A, B, and C.

	A	B	C
Peak, au	123.3 ± 49.9	132.7 ± 47.3	128.7 ± 20.7
FWHM, s	5.8 ± 1.3	6.0 ± 1.1	4.9 ± 0.7
TTP, s	20.0 ± 1.8	19.3 ± 4.2	13.3 ± 1.4
RT, s	4.9 ± 1.1	5.1 ± 1.0	4.1 ± 0.6

**Figure 3.**
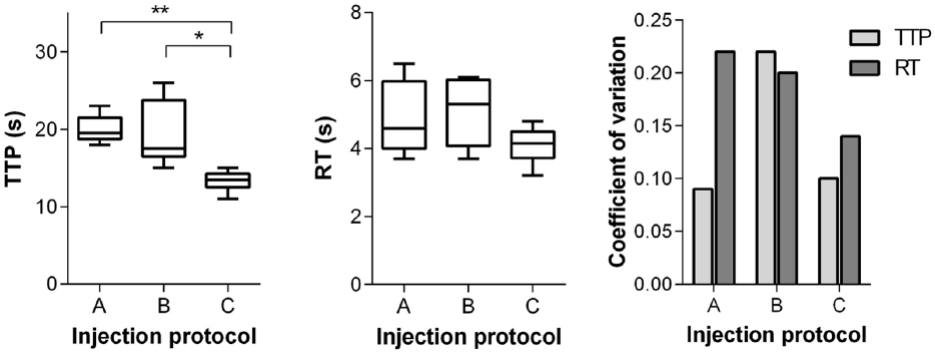
Quantitative evaluation of test bolus timing and parameter variation for injection protocols A, B, and C: time-to-peak (TTP, left), rise time (RT, middle), and coefficient of variation (right). Statistically significant differences are indicated by an asterisk (*p < .05; **p < .001).

The test bolus curve time course showed a significantly shorter TTP for protocol C compared with protocols A (*p* = .016) and B (*p* = .0004). The RT ranged from 4.1 ± 0.6 seconds (protocol C) to 5.1 ± 1.0 seconds (protocol B). The coefficient of variation for TTP was comparably low for protocols A (0.09) and C (0.1) but considerably higher for protocol B (0.22). The RT after automatic injection (protocol C) exhibited lower variability compared with hand injection (Figure 3).

### Quantitative analysis of head and neck MRA

**Figure 4.**
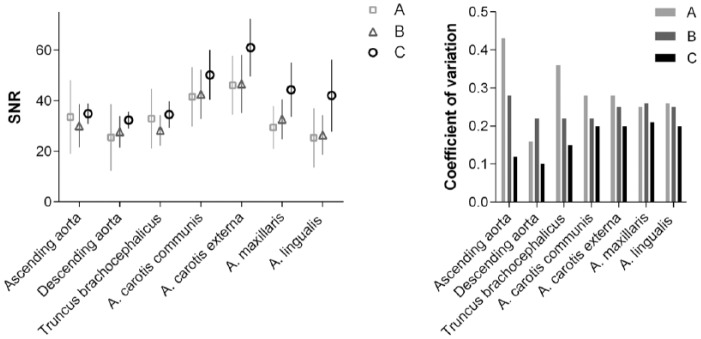
Quantitative signal-to-noise ratio (SNR) evaluation for head and neck magnetic resonance angiography (left) and respective coefficient of variations (right) for injection protocols A (squares/gray), B (triangles/dark gray), and C (circles/black).

Quantitative evaluation in the 7 vascular regions investigated demonstrated a significantly higher SNR for protocol C compared with protocols A (*p* < .0001) and B (*p* < .0001). No significant differences were observed between protocols A and B. Independent of the applied injection protocol, the highest SNR was observed in the carotid arteries (Figure 4, left). The higher SNR for protocol C was most pronounced for the more distal vessels in the head region (A. maxillaris and A. lingualis) than for the large vessels in the thoracic region (aorta and truncus brachiocephalicus). In all vascular regions, the variation in SNR between animals was lower for the automatic injection (protocol C) than for the hand injections (Figure 4, right). Manual injection by 1 technician (protocol A) results in higher signal variability than injection by 6 different technicians.

### Qualitative analysis of head and neck MRA

**Figure 5.**
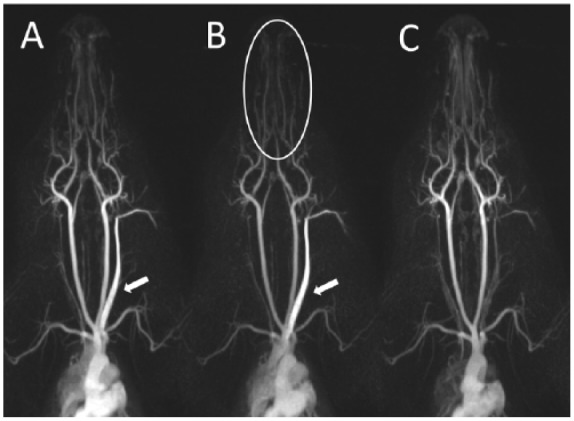
Qualitative evaluation of signal enhancement on maximum intensity projections. Strong enhancement of the contrast-supplying vena subclavian (arrow) and low contrast of the smaller distal vessels of the tongue-jaw region (circle) were observed in some animals with injection protocols A and B.

A qualitative evaluation of signal enhancement was performed using the MIP images. The GBCA was injected into an ear vein, ie, the jugular vein as a contrast-supplying vessel was within the MRA scan range. Strong contrast of the GBCA-supplying jugular vein was found in 50% of the hand injections (protocols A and B, Figure 5). In contrast, no superimposed signal from the V. jugularis was observed from automatic injection (protocol C). Furthermore, visualization of the smaller distal vessels of the tongue (A. sublingualis) and jaw region (A. buccalis, A. palatina, and A. infraorbitalis) was poor in 1 of 6 (3/6) cases for protocol A (B), whereas protocol C showed consistent contrast in all animals (Table 2, Figure 5).

**Table 2.** Qualitative evaluation of vascular contrast in maximum intensity projection images: number of animals for injection protocols A, B, and C that showed high contrast in the contrast agent–supplying vessel (V. jugularis) and low contrast in the smaller vessels in the tongue-jaw region.

	A	B	C
High-contrast V. jugularis	3/6	3/6	0/6
Low-contrast tongue-jaw region	1/6	3/6	0/6

## Discussion

In this study, automatic, injector-based GBCA administration (protocol C) was compared with manual hand injections performed by either 1 experienced technician (protocol A) or 6 less-experienced technicians (protocol B). Test bolus measurements in the carotid artery were used to characterize the vascular bolus shape and timing for both types of contrast injection. Quantitative analysis of the respective bolus shapes showed no statistically significant differences. However, the inter-individual variations in peak signal intensity and bolus width (FWHM) were considerably lower for automatic injection. Thus, GBCA injection with power injectors results in a more predictable and standardized bolus shape in the vascular target region. Based on these results, it is also reasonable to assume higher reproducibility for the automatic contrast injection. The bolus shapes of the 2 different hand injection protocols showed considerable difference in the averaged test bolus curve with large variations for protocol B (6 less-experienced technicians). However, when considering individual bolus shapes, the peak signal and FWHM are comparable for both hand injection protocols. In fact, the difference is the individual TTP in which protocol B showed more than twice the coefficient of variation than protocol A. This finding demonstrates the deviations in operator-dependent injection performance.

Inappropriate bolus timing is a major limitation of CE-MRA.^5,18,21^ The test bolus method reduces the impact of individual patient physiology on bolus timing as the test bolus considers the individual TTP.^17^ Bolus tracking techniques are independent of the bolus travel time from the injection site to the vascular target region and depend only on the signal intensity threshold that triggers the start of the scan (manually or automatically) and the bolus RT. The injector-based contrast protocol had a shorter TTP and RT, but more importantly, it also had a higher degree of standardization compared with the manual injections. This observation is important for both timing techniques, ie, the test bolus method that requires 2 reproducible injections and the bolus tracking that relies on a predefined scan delay between bolus arrival and bolus peak.

In the quantitative MRA signal evaluation, a significantly higher SNR was observed for automatic injection compared with both hand injection protocols. Interestingly, the highest SNR was shown at the location of the test bolus (carotid arteries) independent of the contrast protocol used. Thus, synchronization of the bolus peak and central *k*-space acquisition was generally achieved for all protocols. The largest differences between the hand-based and injector-based protocols were observed for more distal vessels. This result is also reflected in the qualitative contrast analysis of the smaller vessels in the tongue and jaw region. Here, poor contrast was found in 1 and 3 animals for protocols A and B, respectively. This low vascular contrast might be caused by nonoptimal bolus shapes. Although the test bolus shapes for the 3 protocols had no significantly different parameters, it is likely that the bolus shape of the larger main bolus varies to a greater degree. The more compact bolus shape achieved with automatic injection with a small volume test bolus could be enhanced when considering the longer injection time of the main bolus. Our hypothesis that a broader bolus shape results from hand injections is supported by the strong contrast of the GBCA-supplying vessel near the site of injection, which was observed in 50% of the hand injections. Another explanation for the different outcomes may be suboptimal scan timing. The test bolus approach requires 2 reproducible injections, which might be not achieved by manual injection. Interestingly, the SNR of the 2 hand injection protocols revealed comparable results but a higher coefficient of variation for protocol A (1 experienced technician) compared with protocol B (6 less-experienced technicians). Thus, operator-dependent injection performance appears to play a secondary role compared with other effects such as the reproducibility of test and main bolus.

This animal study serves as a model and has limitations compared with clinical situations. The higher heart rate and lower blood volume of the pigs may result in a shorter TTP and narrower bolus shape compared with that in humans. In addition, the body weight of the animals and hence the administered GBCA volumes (0.5 mL test bolus, 2.28 ± 0.36 mL main bolus) were rather low compared with the human situation. Thus, during the injection procedure, the entire GBCA volume remained in the tubing and did not initially enter the body. An effective injection of GBCA into veins is accomplished by the subsequent injection of the saline chaser. Consequently, the impact of the gap between GBCA and saline administration is not reflected in this study. The temporal gap can particularly affect manual injections in which an intrinsic time delay exists when using a 3-way stopcock between the 2 injections. The significantly longer TTP observed for hand injection protocols may reflect this additional time to some extent. For the larger GBCA volumes used in clinical practice, it can be assumed that this temporal gap has a considerable impact on the vascular bolus shape, and hence, MRA image quality as the switching from saline to contrast disrupts the continuous GBCA delivery to the veins. Therefore, specially designed tubing sets for hand injections that facilitate automatic switching from GBCA to saline injections were suggested.^18^

This study investigated the impact of contrast administration methods on 3D-MRA parameters. However, a well-defined bolus shape is also critical for time-resolved 4-dimensional MRA^11,22^ and other emerging dynamic contrast-enhanced MRI techniques such as cardiac perfusion imaging.^23^ The latter also require higher flow rates compared with MRA which might be more difficult to realize with hand injections. In addition to diagnostic image quality, there are several other potential benefits of power injector–based GBCA administration that were not investigated in this study. One example is a simplified workflow, which does not require a physician or technician to be present in the scan room for injection and enables the performance of contrast-enhanced studies with only 1 operator.

In conclusion, this study showed that power injector–based GBCA administration results in a higher degree of standardization of bolus shapes compared with manual injections. This was demonstrated by the lower variation coefficient of the test bolus–derived parameter peak signals FWHM, TTP, and RT. In head and neck MRA, significantly higher and more standardized vascular contrast enhancement was determined after injector-based GBCA administration. More importantly, automatic injection also results in more robust visualization of target vessels and thus a potentially higher diagnostic image quality compared with hand injection. Therefore, the use of MR injectors should be recommended for MRA.
